# Author Correction: Genetic insights into the social organisation of the Avar period elite in the 7th century AD Carpathian Basin

**DOI:** 10.1038/s41598-020-69583-x

**Published:** 2020-08-04

**Authors:** Veronika Csáky, Dániel Gerber, István Koncz, Gergely Csiky, Balázs G. Mende, Bea Szeifert, Balázs Egyed, Horolma Pamjav, Antónia Marcsik, Erika Molnár, György Pálfi, András Gulyás, Bernadett Kovacsóczy, Gabriella M. Lezsák, Gábor Lőrinczy, Anna Szécsényi-Nagy, Tivadar Vida

**Affiliations:** 1 Institute of Archaeology, Research Centre for the Humanities, Hungarian Academy of Sciences Centre of Excellence, 1097 Budapest, Hungary; 2 Department of Genetics, ELTE Eötvös Loránd University, 1117 Budapest, Hungary; 3 Institute of Archaeological Sciences, ELTE Eötvös Loránd University, 1088 Budapest, Hungary; 4 Department of Reference Samples Analysis, Institute of Forensic Genetics, Hungarian Institute for Forensic Sciences, 1027 Budapest, Hungary; 5 Department of Biological Anthropology, University of Szeged, 6726 Szeged, Hungary; 6 Jász Museum, 5100 Jászberény, Hungary; 7 Katona József Museum, 6000 Kecskemét, Hungary; 8 Institute of History, Research Centre for the Humanities, Hungarian Academy of Sciences Centre of Excellence, 1097 Budapest, Hungary; 9 Móra Ferenc Museum, 6720 Szeged, Hungary

Correction to:* Scientific Reports*10.1038/s41598-019-57378-8, published online 22 January 2020

A supplementary file containing Tables S1-S12 was omitted from the original version of this Article. This has been corrected in the HTML version of the Article.

